# Impact of bariatric surgery on depression, anxiety and stress symptoms among patients with morbid obesity: international multicentre study in Poland and Germany

**DOI:** 10.1192/bjo.2021.1084

**Published:** 2022-01-25

**Authors:** Anna Paczkowska, Karolina Hoffmann, Jonas Raakow, Matthias Pross, Rafael Berghaus, Michał Michalak, Wiesław Bryl, Kinga Marzec, Dorota Kopciuch, Tomasz Zaprutko, Piotr Ratajczak, Elżbieta Nowakowska, Krzysztof Kus

**Affiliations:** Department of Pharmacoeconomics and Social Pharmacy, Poznan University of Medical Sciences, Poland; Department of Internal Diseases, Metabolic Disorders and Arterial Hypertension, Poznan University of Medical Sciences, Poland; Department of Surgery, Campus Charité Mitte and Campus Virchow Klinikum, Charité - Universitätsmedizin Berlin, Germany; Department of Surgery, DRK Kliniken Berlin, Germany; Department of Computer Science and Statistics, Poznan University of Medical Sciences, Poland; Department of Internal Diseases, Metabolic Disorders and Arterial Hypertension, Poznan University of Medical Sciences, Poland; Department of Pharmacoeconomics and Social Pharmacy, Poznan University of Medical Sciences, Poland; Department of Pharmacology and Toxicology Institute of Health Sciences, Collegium Medicum, University of Zielona Gora, Poland; Department of Pharmacoeconomics and Social Pharmacy, Poznan University of Medical Sciences, Poland

**Keywords:** Bariatric surgery, conservative treatment for morbid obesity, depression, anxiety, stress

## Abstract

**Background:**

There is a need to investigate how adopting different strategies for treating obesity in different countries in the European Union affects the psychological well-being of patients.

**Aims:**

The aim of this study was to perform a comparative evaluation of psychiatric symptoms (depression, anxiety and stress) in patients undergoing bariatric surgery versus patients receiving conservative treatment for morbid obesity in Poland and Germany.

**Method:**

A multicentre international prospective cohort study with 155 patients who underwent bariatric surgery and 409 patients who received conservative weight reduction treatment. Evaluation of the psychiatric symptoms was carried out for each patient at baseline and after 12 months of active treatment using a standardised Depression Anxiety Stress Scale questionnaire (DASS-21) questionnaire.

**Results:**

After 12 months of active treatment, the level of psychiatric symptoms (depression, anxiety and stress) significantly decreased in both groups of patients: surgically treated versus conservatively treated patients from Poland and also from Germany. The median change in level of psychiatric symptoms among patients from both countries was significantly higher among surgically treated patients compared with conservatively treated patients (Poland *P* < 0.0001; Germany *P* < 0.0001). Improvements in the patient's mental health as a consequence of treatment were dependent on the specific strategy for treating obesity adopted in the analysed countries, the percentage of total weight loss and on gender.

**Conclusions:**

The use of bariatric surgery in both Poland and Germany compared with non-surgical treatment for obesity resulted in more measurable benefits in the form of a decrease in psychiatric symptoms (depression, anxiety and stress) and reduction in body weight.

## Background

Many studies have shown that obesity is associated with a psychopathological burden, and has a negative impact on health-related quality of life.^1^ Meta-analyses have shown cross-cultural differences in the relationship between obesity and mental health. The associations among psychological predictors are related to lower family affluence, self-assessed attractiveness, gender, health policy, life satisfaction and socioeconomic conditions.^2^

Many studies have shown that bariatric surgery compared with non-surgical treatment for obesity leads to more significant weight loss and higher remission of comorbidities.^3,4^ Moreover, some studies indicate that bariatric surgery is generally associated with improved mental health and quality of life and even improvement in the patient's financial situation.^5^ The effect of bariatric surgery on health, including psychological aspects, has been analysed by a few scientists, including Borgeraas et al.^6^ Although bariatric surgery is generally effective, there is great variability in outcome.^7^ The significant predictors influencing improvement in patients’ mental health as a result of bariatric surgery include the type of bariatric surgery performed, weight loss, medical comorbidity and social support. It is also likely that pre-existing and post-surgical physical, psychological and social factors interact with weight loss to influence improvements in mental health following bariatric surgery.^8,9^ In a study by Picot et al improvement in mental health was greater in people who underwent laparoscopic sleeve gastrectomy and gastric bypass surgery compared with laparoscopic adjustable gastric banding (1 year) and vertical banding gastroplasty (2 years). This may be because these procedures result in greater weight loss in this time period.^10^

In Poland, the influence of bariatric surgery on the psychological behaviour of patients has not yet been thoroughly investigated. Moreover, the literature lacks international comparisons of the effectiveness of the obesity treatment strategies adopted in individual countries. So far, a study conducted by Genco et al^11^ showed differences in clinical efficacy and patient satisfaction with bariatric surgery in three European countries (Italy, Belgium and Spain). Psychosocial variables as well as the availability of psychiatric and psychological support predict weight loss or mental health after obesity surgery.^12^ Interesting findings come from a Portuguese case–control study, which aimed to assess the impact of social support on the final outcome of bariatric surgery. The authors confirmed that perceived stronger social support was associated with better mental health and greater weight loss in both the pre- and post-operative groups.^13^ A meta-analysis by Dawes et al revealed that there was conflicting evidence on the association between pre-operative psychopathology and post-operative weight loss.^14^

There is also insufficient scientific data to assess the impact the type of bariatric surgery undergone has on patients’ mental health. Thus, as the number of patients undergoing bariatric surgery increases in high-income countries (such as Poland and Germany), so does the need to investigate how psychiatric symptoms may influence the results of bariatric surgery compared with conservative treatment for obesity.

Moreover, the obesity treatment strategy developed by the German healthcare system differs significantly from that developed in Poland. In Germany, specialised obesity treatment health centres that offer comprehensive conservative treatment focus on four areas of intervention and application: advice on diet and training, movement therapy and exercise, psychoeducation and behavioural therapy interventions.^15^ Whereas in Poland, the obesity treatment process is carried out as part of primary healthcare or in a few specialist out-patients obesity clinics.^16^ There is a need to investigate how the strategy adopted for treating obesity in different countries of the European Union affects the psychological well-being of patients.

## Aims

This study aimed to perform a comparative evaluation of the percentage of weight loss and changes in psychiatric symptoms (depression, anxiety and stress) in patients undergoing bariatric surgery versus non-surgical treatment (conservative treatment) for morbid obesity in Poland versus Germany.

## Method

### Study population

A multicentre international prospective cohort study was conducted at four selective healthcare facilities in Germany and Poland. The target group of 564 patients consists of two groups of patients: patients undergoing bariatric surgery – 155 patients (73 from Germany and 82 from Poland) and patients receiving conservative weight reduction treatment – 409 patients (137 from Germany and 272 from Poland).

The inclusion criteria for both groups were: age ≥ 18 years; surgically or conservatively treated obesity in the study time horizon – from 1 January 2019 to 31 December 2019; body mass index (BMI) ≥ 35 kg/m^2^ associated with comorbidities; stable obese for at least 5 years; absence of illicit drugs or alcohol misuse; lack of moderate or severe psychosis or dementia; and patient's consent to participate in the study. Their attending physician decided to include a patient in the study based on the inclusion and exclusion criteria defined above.

### Obesity treatment received

Surgical or endoscopic procedures were performed using the German and Polish guidelines for bariatric surgery.^17,18^

In Germany, the conservative treatment offered was a 1-year multimodal out-patient weight reduction programme. This conventional out-patient weight reduction treatment consisted of four areas of intervention and application:

- advice on diet and training;
- movement therapy and training;
- psychoeducation; and
- behavioural therapy interventions.

These interventions were conducted in group settings designed for eight to ten participants and were held twice a week for 2 h during the first 6 months and once weekly for 3 h during the second 6 months. This programme was led by a physician and clinical psychologist.

In Poland, the conservative treatment was carried out in a specialist obesity out-patient clinic. Conventional out-patient weight reduction treatment was led by a physician and dieticians who provided regular (on average once a month) individual medical consultations on the principles of proper nutrition, lifestyle changes, pharmacotherapy and the selection of an appropriate low-calorie diet.

Before the study, each eligible study participant was informed about the study objectives and conditions and gave their written informed consent to participate in the study. All procedures involving human patients were approved by the Ethics Committee of the Poznan University of Medical Sciences and by the Ethics Committee of the Charité - Universitätsmedizin Berlin.

### Study technique

Evaluation of the psychiatric symptoms (depression, anxiety and stress) in the participants who underwent bariatric surgery or conservative treatment for morbid obesity was carried out using a standardised Depression Anxiety Stress Scale questionnaire in Polish and German, version (DASS–21).^19^ The DASS-21 is a 21-item questionnaire that is commonly used as a tool for the assessment of the levels of self-perceived depression, anxiety and stress in participants aged 14 years and above. The DASS-21 was constructed as a shorter version of the DASS-42 questionnaire and has three main domains: depression, anxiety and stress. In each domain there are seven questions, with a Likert scale ranging from zero to three. Polish and German versions of this questionnaire were adequately translated with high validity and internal reliability.^20–22^

During the 12 months of active treatment, each participant completed the questionnaires twice. Non-surgical patients in Germany completed the questionnaires before (at baseline) and after completing the 1-year multimodal out-patient weight reduction programme (the DASS-21 was administrated twice: on the first and last visit). The completion rate during the study time horizon was 96%. Similarly, non-surgical patients in Poland completed the questionnaires at baseline and after 1 year of regular medical and dietary visits. Among the surgical patients, the DASS-21 was administered 1 day before surgery (at baseline) and 1 year after their bariatric surgery.

In addition, specific information about patients was collected from medical records (the duration of the disease, the course of treatment to date, the presence of comorbidities). All the patients completed the questionnaires independently. Completed questionnaires (100% filled out by the patients) were included in the study analysis.

At the beginning and the end of the study, a physical examination was undertaken, and updated patient medical records were analysed. Weight, height and BMI was assessed in the whole study group at baseline and after the 12 months of active treatment. The weight in kilogram was analysed using electric scales (TANITA TBF-240, Arlington Heights, USA). Height was measured using a standard measuring rod, barefoot and in the Frankfort position. To evaluate the efficacy of the treatments, we calculate %TWL (percentage of total weight loss) and %EWL (percentage of excess weight loss). The formulas proposed by Baltasar et al^23^ were used to calculate the BMI, %TWL, and %EWL as follows:

- BMI: weight (kg)/height (m^2^);
- %TWL: [ (initial weight − current weight)/ (initial weight)] × 100;
- %EWL: [ (initial weight − current weight)/ (initial weight – ideal weight)] × 100.

%EWL variables were created, with the ideal weight taken from the tables for the Polish^24^ and Germany population.^25^

### Statistical analysis

Results were analysed statistically. The quantitative parameters were presented using mean value, median and standard deviation. The results between analysed groups were compared using Student's t-test. In cases where data did not follow the normal distribution (Shapiro–Wilks test), comparison was performed using the Mann–Whitney test. The comparison of more than two groups was performed using Kruskal–Wallis test with *post hoc* Dunn's test. The chi-square test for independence was used to analyse categorical data. The relationship between the changes in levels of psychiatric symptoms (depression, anxiety and stress) and analysed parameters was performed by multiple regression analysis. For categorical data, the coefficients for a specified level were compared with the reference level.

The analysis was performed with the use of statistical package TIBCO Software Inc. (2017). Statistica (data analysis software system), version 13. http://statistica.io. All tests were considered significant at *P* < 0.05.

## Results

### Study groups characteristics

The sociodemographic characteristics of the sample are shown in Table 1. The target group of 564 patients consists of two groups of patients:

- patients undergoing bariatric surgery – 155 patients (73 from Germany and 82 from Poland);
- and patients receiving conservative weight reduction treatment – 409 patients (137 from Germany and 272 from Poland).

**Table 1:** Baseline demographic and clinical characteristics of patients with obesity from Poland and Germany (*n* = 564)

	Poland	Germany	*P*
Group size			
Total	354	210	
Female *n* (%)	274 (77.40)	158 (75.24)	0.7563
Male *n* (%)	80 (22.60)	52 (24.76)	0.5369
Surgically treated patients *n* (%)	82 (23.16)	73 (34.76)	
Conservatively treated patients *n* (%)	272 (76.84)	137 (65.24)	
Age, years: total mean (s.d.)^a^	45.20 (15.69)	45.70 (9.70)	0.8541
Body mass index			
Body mass index, total mean (s.d.)^a^	36.92 (8.12)	36.87 (10.06)	0.9452
I degree of obesity [30–34.9 kg/m^2^], *n* (%)	99 (27.96)	57 (27.14)	0.8876
II degree of obesity [35–39.9 kg/m^2^], *n* (%)	105 (29.66)	53 (25.10)	0.0681
III degree of obesity [≥40 kg/m^2^], *n* (%)	150 (42.38)	100 (47.76)	0.0741
Duration of the disease, years: total mean (s.d.)^a^	17.67 (11.61)	17.00 (10.51)	0.7561
Vocational education, *n* (%)			
Low level	129 (36.44)	77 (36.71)	0.8941
Average level	151 (42.66)	86 (41.06)	0.7452
High level	74 (20.90)	47 (22.23)	0.1237
Material status, *n* (%)^b^			
Definitely good	36 (10.20)	22 (10.50)	0.9456
Good	122 (34.36)	75 (35.66)	0.5632
Average	154 (43.62)	93 (44.16)	0.6569
Bad	27 (7.63)	15 (7.11)	0.8962
Definitely bad	15 (4.19)	5 (2.57)	0.0956
Type of bariatric surgery, *n* (%)			
Gastric balloon	1 (1.22)	0.00	
Laparoscopic adjustable gastric banding	5 (6.10)	0.00	
Laparoscopic Roux-en-Y gastric bypass	9 (10.98)	40 (54.79)	<0.001
Laparoscopic sleeve gastrectomy	67 (81.70)	33 (45.21)	<0.001
Comorbidities, *n* (%)			
Type 2 diabetes mellitus	96 (27.11)	60 (28.57)	0.4589
Hypertension	161 (45.48)	100 (47.61)	0.1253
Dyslipidaemia	63 (17.79)	40 (19.04)	0.1478
Hyperuricemia	14 (3.95)	12 (5.71)	0.0635
Metabolic syndrome	48 (13.56)	35 (16.66)	0.0856
Coronary artery disease	88 (24.85)	52 (24.76)	0.7563

a. Statistically significant difference: Germany versus Poland for *P* < 0.05.

b. Material status categories:

Definitely good >3000 EUR per month

Good 2001–3000 EUR per month

Average 1001–2000 EUR per month

Bad 500–1000 EUR per month

Definitely bad <500 EUR per month

Among surgical patients who underwent bariatric surgery in Poland, 1 (1.22%) of patients received a gastric balloon, 5 (6.10%) received laparoscopic adjustable gastric banding, 9 (10.98%) received laparoscopic Roux-en-Y gastric bypass and 67 (81.70%) underwent laparoscopic sleeve gastrectomy. Among surgical patients who underwent bariatric surgery in Germany, 40 (54.79%) received a laparoscopic Roux-en-Y gastric bypass and 33 (45.21%) underwent laparoscopic sleeve gastrectomy.

In the groups of patients from both Germany and Poland, most patients were female – 75.24% and 77.40%, respectively. The average age of the study participant was 45.70 (s.d. = 9.70) years in the German group and 45.20  (s.d. = 15.69) in the Polish group. The mean BMI in the German group was 36.87  (s.d. = 10.06) and in the Polish group 36.92  (s.d. = 8.12). There were no significant differences in terms of gender, age, baseline BMI, degree of obesity, duration of the disease, level of education and material status between the two groups (Table 1).

### Evaluation of the psychiatric symptoms (depression, anxiety and stress) of patients undergoing bariatric surgery or conservative treatment for morbid obesity in Poland and Germany.

A comparative analysis using the Wilcoxon matched pairs test showed that at baseline, patients from both groups (Poland versus Germany) did not significantly differ regarding levels of depression; anxiety; and stress symptoms according to DASS-21 questionnaire (21 Poland versus 22 in Germany, *P* = 0.4366; 16 in Poland versus 17 in Germany, *P* = 0.7213; 22 in Poland versus 23 in Germany, *P* = 0.6356, respectively) (Table 2).

**Table 2:** Evaluation of the psychiatric symptoms (depression, anxiety and stress) of patients undergoing bariatric surgery or conservative treatment for morbid obesity in Poland and Germany (*n* = 564)

	Baseline				Follow-up			
Outcome parameters	General	Surgically treated patients	Conservatively treated patients	*P*	General	Surgically treated patients	Conservatively treated patients	*P*
Level of depression, median (Q_1_ [lower quartile]–Q_3_ [upper quartile])								
Poland	21 (16−27)	21 (18−24)	21 (16−28)	0.3016^c^	14 (8−24)	6 (4−12)	18 (12−28)	<0.0001^c^
Germany	22 (18−29)	22 (18−28)	23 (18−30)	0.7358^c^	12 (4−24)	4 (0−9)	20 (10−28)	<0.0001^c^
*P*	0.4366^b^	0.4893^d^	0.1351^d^	−	0.0001^a^	0.0342^d^	0.5432^d^	
Level of anxiety, median (Q_1_–Q_3_)								
Poland	16 (10−20)	15 (14−19)	15 (10−20)	0.1014^c^	10 (4−18)	4 (0−8)	12 (6−18)	<0.0001^c^
Germany	17 (12−22)	16 (16−26)	16 (10–20)	0.3584^c^	8 (4−16)	4 (0−7)	10 (6−20)	<0.0001^c^
*P*	0.7213^b^	0.0839^d^	0.1439^d^		<0.0001^a^	0.7193^d^	0.6100^d^	
Level of stress, median (Q_1_–Q_3_)								
Poland	22 (15−26)	22 (18−26)	22 (15−26)	0.2254^c^	16 (8−24)	8 (4−14)	18 (12−26)	<0.0001^c^
Germany	23 (18−30)	22 (18−31)	23 (18−30)	0.7322^c^	16 (6−26)	8 (2−14)	20 (14−28)	<0.0001^c^
*P*	0.6356^b^	0.2443^d^	0.2961^d^		0.8671^a^	0.6444^d^	0.0422^d^	

a. Wilcoxon matched pairs test, level of depression, anxiety and stress at baseline versus after follow-up in Poland and Germany.

b. Mann-Whitney *U*-test, change in levels of depression, anxiety and stress in Poland versus in Germany.

c. Mann-Whitney *U*-test, a comparison of the psychiatric symptoms (depression, anxiety and stress) of patients who underwent bariatric surgery versus conservative treatment in Poland and Germany.

d. Mann-Whitney *U*-test, a comparison of the psychiatric symptoms (depression, anxiety and stress) of patients in Poland versus in Germany regarding the methods of obesity treatment (bariatric, conservative).

Also, a comparative analysis using the Mann–Whitney *U*-Test revealed that surgically treated patients from Poland and Germany did not significantly differ concerning levels of depression, anxiety and stress symptoms compared with conservatively treated patients (Table 2). Similarly, at baseline, depression, anxiety and stress levels among surgically treated patients from Poland did not show significant differences compared with surgically treated patients from Germany. The same situation was observed in the case of conservatively treated patients from Poland compared with Germany (Table 2).

### Depression symptoms

The analysis of data using the Mann–Whitney *U*-test revealed that after 12 months of active treatment, the general level of depression symptoms significantly decreased from 21 to 14 (Table 2) (median decrease 3, *P* < 0.0001) among Polish patients and showed a highly significant reduction from 22 to 12 (median decrease 7, *P* < 0.0001) in the Germany patients. A significantly greater change in the level of depression was observed among patients from Germany (*P* = 0.0005) (Table 3).

**Table 3:** Evaluation of the psychiatric symptoms (depression, anxiety and stress) of patients undergoing bariatric surgery or conservative treatment for morbid obesity in Poland and Germany (*n* = 564)

	Change			
Outcome parameters	General	Surgically treated patients	Conservatively treated patients	*P*
Level of depression, median (Q_1_ [lower quartile]–Q_3_ [upper quartile])				
Poland	3 (0 to 7)	12 (7 to 19)	2 (0 to 4)	<0.0001^a^
Germany	7 (0 to 15)	18 (10 to 25)	1 (0 to 8)	<0.0001^a^
*P*	0.0005^b^	0.0065^c^	0.3379^c^	
Level of anxiety, median (Q_1_–Q_3_)				
Poland	3 (0 to 8)	11 (7 to 14)	2 (0 to 5)	<0.0001^a^
Germany	6 (0 to 14)	15 (10 to 21)	2 (−1 to 7)	<0.0001^a^
*P*	<0.0001^b^	0.0236^c^	0.2554^c^	
Level of stress, median (Q_1_–Q_3_)				
Poland	3 (0 to 8)	12 (8 to 18)	1 (0 to 4)	<0.0001^a^
Germany	6 (0 to 12)	14 (9 to 20)	1 (−2 to 6)	<0.0001^a^
*P*	0.0133^b^	0.1293^c^	0.8723^c^	

a. Mann-Whitney *U*-test A comparison of the psychiatric symptoms (depression, anxiety and stress) of patients underwent bariatric surgery versus conservative treatment in Poland and Germany.

b. Mann-Whitney *U*-test Change of level of depression, anxiety and stress in Poland *v*. in Germany.

c. Mann-Whitney *U*-test A comparison of the psychiatric symptoms (depression, anxiety and stress) of patients from Poland versus in Germany regarding with the methods of obesity treatment (bariatric, conservative).

The analysis of data using the Mann–Whitney *U*-test revealed that after 12 months of active treatment, among surgically treated patients from both countries, levels of depression symptoms significantly decreased (Poland from 21 to 6, median change 12; Germany from 22 to 4, median change 18). The median change was significantly higher among patients from Germany (*P* = 0.0065) (Table 3).

Among conservatively treated patients from both countries after 12 months of active treatment there was a considerable decrease in the level of symptoms of depression (Poland from 21 to 18, median change 2; Germany from 23 to 20, median change 1). There were no significance differences in median change between both groups (Poland compared with Germany).

Analysis of data using the Mann–Whitney *U*-test revealed that the median shift in the level of depression symptoms among patients from Poland and also from Germany was significantly higher among surgically treated patients compared with conservatively treated patients (Tables 2 and 3).

### Anxiety symptoms

Similarly, after the 12 months of active treatment, the general level of anxiety symptoms significantly decreased from 16 to 10 (median decrease 3, *P* < 0.0001) among Polish patients and showed a highly significant reduction from 17 to 8 (median decrease 6, *P* < 0.0001) in the Germany patients. A significantly greater change in the level of anxiety was observed among the group of patients from Germany (*P* < 0.0001).

Analysis of data using the Mann- Whitney *U*-test revealed that after 12 months of active treatment, among surgically treated patients from both countries, the level of anxiety symptoms significantly decreased (Poland from 15 to 4, median change 11; Germany from 16 to 4, median change 15). Median change was significantly higher among patients from Germany.

Among conservatively treated patients from both countries after the 12 months of active treatment it was observed that there was a significantly decreased level of anxiety symptoms (Poland from 15 to 12, median change 2; Germany from 16 to 10, median change 2). There were no significance differences in median change between both groups (Poland compared with Germany).

Analysis of data using the Mann–Whitney *U*-test revealed that the median shift in the level of anxiety symptoms among patients from Poland and also from Germany was significantly higher among surgically treated patients compared with conservatively treated patients (Tables 2 and 3).

### Stress symptoms

Similarly, after 12 months of active treatment, general levels of stress symptoms significantly decreased from 22 to 16 (median decrease 3, *P* < 0.0001) among Polish patients and showed a highly significant reduction from 23 to 16 (median decrease 6, *P* < 0.0001) in the German group of patients. Median change in general stress levels did not have significant differences between the analysed group of patients (Tables 2 and 3). The analysis of data using the Mann–Whitney *U*-test revealed that after the 12 months of active treatment, among surgically treated patients from both countries, the level of stress symptoms significantly decreased (Poland from 22 to 8, median change 12; Germany from 22 to 8, median change 14). There were no significance differences in median change between both groups (Poland compared with Germany).

Among conservatively treated patients from both countries after the 12 months of active treatment a significantly decreased level of stress symptoms (Poland from 22 to 18, median change 1; Germany from 23 to 20, median change 1) was observed. There were no significance differences in median change between both groups (Poland compared with Germany). The analysis of data using the Mann–Whitney *U*-test revealed that the median shift in levels of stress symptoms among patients from Poland and also from Germany was significantly higher among surgically treated patients compared with conservatively treated patients (Tables 2 and 3).

### The impact of the type of bariatric surgery performed on patients’ mental health

The analysis undertaken show no statistically significant differences in the level of perceived depression, anxiety and stress in both the Polish and German patients treated for obesity, depending on the type of bariatric surgery performed (*P* > 0.05). (Supplementary Table 1 available at https://doi.org/10.1192/bjo.2021.1084).

### Multiple regression model for confounders.

Additional multiple regression analysis that was performed for confounders that may influence the level of psychiatric symptoms outcome (depression, anxiety and stress) in both groups showed that change in depression symptoms levels were significantly associated with the country, and %TWL. Patients from Germany and with a higher %TWL had the highest decrease in depression symptoms. It was found that change in anxiety symptom levels were significantly associated with the country, gender and %TWL. Patients from Germany, men and with those with higher %TWL had the highest decrease in anxiety symptoms. Similarly, changes in stress symptom levels were significantly associated with country, gender and %TWL. Patients from Germany, men and those with higher %TWL had the highest decrease in stress symptoms. Age, BMI classification, financial situation, and the number of specialists involved in the treatment process do not influence the patient's level of psychiatric symptoms (Table 4, Supplementary Table 2).

**Table 4:** Multiple regression model for the psychiatric symptoms outcomes (depression, anxiety and stress) among patients with morbid obesity from Poland and Germany

	Level of depression		Level of anxiety		Level of stress	
Variable	Coefficient (95% CI)	*P*	Coefficient (95% CI)	*P*	Coefficient (95% CI)	*P*
Country						
Germany	(reference)		(reference)		(reference)	
Poland	−2.14 (−3.01 to −1.27)	<0.0001	−2.70 (−3.52 to −1.88)	<0.0001	−1.35 (−2.34 to −0.35)	0.008
Gender						
Female	(reference)		(reference)		(reference)	
Male	0.74 (−0.17 to 1.66)	0.111	1.54 (0.68 to 2.41)	<0.0001	1.53 (0.49 to 2.58)	0.004
Age	−0.007 (−0.03 to 0.02)	0.604	0.006 (−0.02 to 0.03)	0.651	0.02 (−0.008 to 0.05)	0.157
Body mass index classification						
Normal body mass	(reference)		(reference)		(reference)	
Overweight	1.24 (−0.91 to 3.41)	0.258	0.88 (−1.15 to 2.92)	0.395	−1.02 (−3.49 to 1.44)	0.414
I degree of obesity [30–34.9 kg/m^2^]	0.74 (−1.18 to 2.67)	0.449	1.31 (−0.51 to 3.13)	0.158	−1.51 (−3.72 to 0.68)	0.177
II degree of obesity [35–39.9 kg/m^2^]	1.16 (−0.79 to 3.12)	0.244	1.30 (−0.54 to 3.15)	0.167	−1.10 (−3.34 to 1.13)	0.332
III degree of obesity [≥40 kg/m^2^]	0.78 (−1.14 to 2.70)	0.426	0.60 (−1.21 to 2.41)	0.516	−1.13 (−3.33 to 1.05)	0.309
Financial situation						
Definitely good	(reference)		(reference)		(reference)	
Good	0.75 (−0.42 to 1.92)	0.209	−0.41 (−1.52 to 0.69)	0.460	0.46 (−0.87 to 1.80)	0.498
Mean	0.81 (−0.37 to 2.00)	0.177	−0.76 (−1.89 to 0.69)	0.179	−0.13 (−1.48 to 1.22)	0.848
Bad	1.03 (−0.52 to 2.60)	0.194	−0.57 (−2.05 to 0.90)	0.445	−0.29 (−2.07 to 1.49)	0.750
Definitely bad	1.54 (−1.15 to 4.24)	0.261	0.32 (−2.22 to 2.87)	0.805	1.55 (−1.52 to 4.63)	0.322
Percentage of total weight loss	0.66 (0.62 to 0.69)	<0.0001	0.54 (0.51 to 0.58)	<0.0001	0.58 (0.54 to 0.62)	<0.0001
The number of specialists involved in the treatment process	−0.43 (−1.02 to 0.15)	0.146	0.41 (−0.14 to 0.96)	0.145	0.25 (−0.41 to 0.92)	0.461

### Efficacy of treatment methods adopted for morbid obesity

Among surgically treated patients from Poland, the median %TWL and %EWL was 26 and 64, respectively (Table 5). Compared with conservatively treated patients from Poland, median %TWL and %EWL were 5 and 11, respectively. The analysis of data using the Mann–Whitney *U*-test revealed that median %TWL and %EWL were significantly higher among surgically treated patients compared with conservatively treated patients (*P* < 0.0001). Similarity among surgically treated patients from Germany median %TWL and %EWL were 28 and 67, respectively. In conservatively treated patients from Germany, median %TWL and %EWL were 7 and 14, respectively. Analysis of data using the Mann–Whitney *U*-test revealed that median %TWL and %EWL were significantly higher among surgically treated patients compared with conservatively treated patients (*P* < 0.0001). Additionally, the Mann–Whitney *U*-test analysis revealed no significant difference in efficacy of the treatment pathways for morbid obesity between both the two groups (Poland versus Germany).

**Table 5:** Evaluation of the percentage of weight loss of patients undergoing bariatric surgery or conservative treatment for morbid obesity in Poland and Germany *n* = 564.

Outcomes	Poland *n* = 354			Germany *n* = 210			*P*, conservatively treated patients in Poland versus Germany	*P*, surgically treated patients in Poland versus Germany
	Surgically treated patients	Conservatively treated patients	*P*	Surgically treated patients	Conservatively treated patients	*P*		
Percentage of total weight loss, median (Q_1_–Q_3_)	26 (22−30)	5 (2−10)	<0.0001^a^	28 (18−37)	7 (2−10)	<0.0001^a^	0.1329^b^	0.6822^b^
Percentage of excess weight loss, median (Q_1_–Q_3_)	64 (60−68)	11 (7−19)	<0.0001^a^	67 (63−75)	14 (10−18)	<0.0001^a^	0.0849^b^	0.4852^b^

a. Mann-Whitney *U*-test, a comparison of the percentage of weight loss of patients who underwent bariatric surgery versus conservative treatment for morbid obesity in Poland and Germany.

b. Mann-Whitney *U*-test, a comparison of the efficacy of treatment methods adopted for morbid obesity within the countries.

## Discussion

### Interpretation of our findings and comparison with findings from other studies

The research results show that the general change in levels of depression, anxiety and stress symptoms because of the method selected for treatment of morbid obesity was significantly greater in patients who were obese living in Germany compared with those in Poland. The general level of psychiatric symptoms after 12 months of active treatment were therefore significantly lower among participants in Germany compared with patients in Poland.

The above situation may result from the fact that there are support groups for patients who are obese in the German obesity treatment centres where the study was conducted. The patient support group for obesity operating at the Charité Hospital in Berlin and the support group working at the Deutsches Rotes Kreuz Hospital in Berlin meet once a month. During the meetings, numerous training sessions are held with experts from various specialties who provide help and support at different stages of obesity treatment.^26^

A systematic review and the results of a meta-analyses carried out by Amiri & Behnezhad^27^ have shown that anxiety disorders and depressive symptoms occur significantly more often in people who are obese and overweight than among people with a healthy body weight. As a result of the considerably higher level of perceived depressive symptoms among Polish patients, it would be advisable to implement psychological/behavioural therapy as an element of comprehensive obesity therapy. The ‘Obesity Balance’ therapeutic programme, implemented at the Charité Hospital, also includes psychoeducation on changing patients’ behaviour and learning to cope with various emotional situations (anxiety, fear, depression).^26^ Psychoeducation undoubtedly reduces the perceived levels of stress, anxiety and depression among patients who are obese and at risk of psychological disorders.

The results of our study conducted in Poland and Germany indicate that bariatric surgery is an effective method for treating obesity. The impact of bariatric surgery on mental health is significantly greater than that from conservative treatment of obesity, in both groups of test participants. The results obtained are consistent with those of other authors.^28^ Similar results were obtained in a study conducted by Buddeberg-Fischer et al.^29^ The study investigated the physical and psychosocial outcomes of patients who underwent bariatric surgery and those who had not. The surgical group achieved a significantly better result for depression, health perception, social interaction and psychosocial functioning than the conventional treatment group.

Our study results have shown that the quality of mental health among patients with morbid obesity depends significantly on gender and the percentage of total weight loss that has been achieved as a result of treatment. Our results are confirmed by the scientific literature. Most scientific data have shown that improvement in a patient's mental health is likely attributed to weight loss and resultant gains relating to body image, self-esteem and self-concept.^3^ Our study results indicate unequivocally that symptoms of depression and anxiety occur to a greater extent among women who are obese than among men. The above situation applies both to people who are obese and who undergo bariatric surgery and those who receive conservative treatment.

Many clinical trials found that obesity is associated with depression;^30,31^ the relationship between depression and obesity is likely affected by several confounding factors. One factor that may influence the relationship between obesity and depression is gender. Carpenter et al^32^ reported that obesity in women was associated with a 37% increase in the prevalence of major depression. Among men, obesity was associated with a decrease in the prevalence of major depression.

Our study results did not indicate that the type of bariatric surgery performed influenced the perceived levels of depression, anxiety and stress symptoms in patients with obesity from Poland and Germany. In a prospective randomised double-blind trial conducted by Murphy et al,^33^ that compared outcomes including weight loss, quality of life, anxiety and depressive symptoms, post-operative complications and mortality after undergoing laparoscopic sleeve gastrectomy versus laparoscopic Roux-en-Y gastric bypass, it was shown that despite significantly greater weight loss after laparoscopic Roux-en-Y gastric bypass, there were no significant differences in the extent of improvements in either quality of life or in anxiety or depression symptoms between the two types of surgery at 1 year. Our results and the results obtained by Murphy et al^33^ are consistent with the results of two previous randomised studies evaluating standard laparoscopic Roux-en-Y gastric bypass and laparoscopic sleeve gastrectomy outcomes among patients with obesity and type 2 diabetes.^34,35^

The results of our research showed unequivocally that in both countries, the effectiveness of reducing total and excess body weight was significantly higher with surgical treatment compared with conservative treatment. The obtained results are in parallel with scientific literature. A metal-analysis carried out by Cheng et al^36^ based on 25 randomised trials, comprising 1194 participants, revealed a significant advantage among surgical patients rather than those receiving non-surgical treatments (*P* < 0.05) in terms of primary end-points (weight loss and diabetic remission). Moreover, bariatric surgery was superior to conventional treatment in terms of secondary metabolic parameters. By mechanically altering the physiological mode of gastrointestinal absorption, bariatric surgery triggers a remarkable decline in excessive weight, hyperglycaemia, cardiovascular risk and correlative mortality compared with conservative treatment.^37^ Therefore, the updated National Institute for Health and Care Excellence guidance (CG189) in the UK has broadened the indications that as a second-line option, all patients with a BMI >30 should be assessed for bariatric surgery following a lack of response to non-surgical interventions.^38^

### Strengths and limitations

The results of our study are innovative as they present one of the first international research reports in the scientific literature of a comparative assessment of the impact of the most commonly used methods for treating obesity (bariatric surgery and conservative treatment) on patient's mental health. In particular, there is a shortage of sufficient research in the field from Poland given the increasing amount of bariatric surgery being performed there. Moreover, the presented data constitute the first scientific report with a comparative analysis of the impact of obesity treatment strategies on patients’ mental health in individual European Union countries.

Our study also has some limitations. The most important limitation is that this study sample was recruited from only four international centres from Poland and Germany. It would be very interesting to roll the study out to other centres, for example outside the European Union. Another significant limitation is the relatively short follow-up time of 1 year caused by the COVID-19 pandemic. The length of follow-up between studies varied greatly, examine improve quality of life as an outcome of bariatric surgery in obese adults, ranging from 1 month to 10 years.^7^ Generally, studies with a shorter follow-up period are more likely to show significant improvements in mental health. Those with more extended follow-up periods generally demonstrated maintenance of early mental health improvements.^39^

In conclusion, bariatric treatment for obesity contributes significantly to an improvement in patients’ mental health both in terms of reducing the levels of their depression, anxiety and stress symptoms and reducing body weight compared with a conservative treatment option for obesity. Improvements in mental health as a consequence of treatment were dependent on the specific strategy for treating obesity adopted in the analysed countries, the percentage of total weight loss and on the gender of the patient and it was not dependent on the type of bariatric surgery, their age, BMI, the patient's financial situation or on the number of specialists involved in the treatment process.

## Data Availability

The data that support the findings of this study are available on request from the corresponding author: aniapaczkowska@ump.edu.pl

## Supplementary material

For supplementary material accompanying this paper visit http://doi.org/10.1192/bjo.2021.1084. click here to view supplementary material
