# Supplementary material for: Impact of bariatric surgery on depression, anxiety and stress symptoms among patients with morbid obesity: international multicentre study in Poland and Germany
Source: BJPsych Open. 2022 Jan 25;8(1):e32. doi: 10.1192/bjo.2021.1084 (PMC8811782; doi:10.1192/bjo.2021.1084)
Supplement: Supplementary file 1 [file S205647242101084Xsup001.docx]

**Supplementary Table 1 Evaluation of the psychiatric symptoms (depression, anxiety and stress) depending on the type of bariatric surgery performed for morbid obesity in Poland and Germany n=156**

| **Level of depression:** | **POLAND n=83** | | | | **GERMANY n=73** | | | |
| --- | --- | --- | --- | --- | --- | --- | --- | --- |
|  | **Type of bariatric surgery** | | | | **Type of bariatric surgery** | | | |
|  | **Gastric balloon (%)** | **Laparoscopic adjustable gastric banding (%)** | **Laparoscopic Roux-en-Y Gastric Bypass (%)** | **Laparoscopic Sleeve Gastrectomy (%)** | **Gastric balloon (%)** | **Laparoscopic adjustable gastric banding (%)** | **Laparoscopic Roux-en-Y Gastric Bypass (%)** | **Laparoscopic Sleeve Gastrectomy (%)** |
| Normal | 0.00 | 60.00 | 60.00 | 69.57  p=0.237 | No applied | No applied | 82.05 | 66.67  p=0.289 |
| Mild | 0.00 | 0.00 | 10.00 | 15.94  p=0.237 |  |  | 7.69 | 18.18  p=0.289 |
| Moderate | 100.00 | 40.00 | 30.00 | 14.49  p=0.237 |  |  | 10.26 | 15.15  p=0.289 |
| Severe | 0.00 | 0.00 | 0.00 | 0.00 |  |  | 0.00 | 0.00 |
| Extremely severe | 0.00 | 0.00 | 0.00 | 0.00 |  |  | 0.00 | 0.00 |
| **Level of anxiety:** | **Gastric balloon (%)** | **Laparoscopic adjustable gastric banding (%)** | **Laparoscopic Roux-en-Y Gastric Bypass (%)** | **Laparoscopic Sleeve Gastrectomy (%)** | **Gastric balloon (%)** | **Laparoscopic adjustable gastric banding (%)** | **Laparoscopic Roux-en-Y Gastric Bypass (%)** | **Laparoscopic Sleeve Gastrectomy (%)** |
| Normal | 100.00 | 60.00 | 60.00 | 68.12  p=0.150 | No applied | No applied | 71.79 | 78.79  p=0.230 |
| Mild | 0.00 | 40.00 | 0.00 | 11.59  p=0.150 |  |  | 5.13 | 0.00  p=0.230 |
| Moderate | 0.00 | 0.00 | 30.00 | 11.59  p=0.150 |  |  | 15.38 | 18.18  p=0.230 |
| Severe | 0.00 | 0.00 | 0.00 | 8.70  p=0.150 |  |  | 7.69 | 0.00  p=0.230 |
| Extremely severe | 0.00 | 0.00 | 10.00 | 0.00  p=0.150 |  |  | 0.00 | 3.03  p=0.230 |
| **Level of stress:** | **Gastric balloon (%)** | **Laparoscopic adjustable gastric banding (%)** | **Laparoscopic Roux-en-Y Gastric Bypass (%)** | **Laparoscopic Sleeve Gastrectomy (%)** | **Gastric balloon (%)** | **Laparoscopic adjustable gastric banding (%)** | **Laparoscopic Roux-en-Y Gastric Bypass (%)** | **Laparoscopic Sleeve Gastrectomy (%)** |
| Normal | 100.00 | 60.00 | 70.00 | 81.16  p=0.426 | No applied | No applied | 76.92 | 81.82  p=0.920 |
| Mild | 0.00 | 0.00 | 0.00 | 5.80  p=0.426 |  |  | 5.13 | 3.03  p=0.920 |
| Moderate | 0.00 | 40.00 | 20.00 | 5.80  p=0.426 |  |  | 12.82 | 9.09  p=0.920 |
| Severe | 0.00 | 0.00 | 10.00 | 7.25  p=0.426 |  |  | 5.13 | 6.06  p=0.920 |
| Extremely severe | 0.00 | 0.00 | 0.00 | 0.00 |  |  | 0.00 | 0.00 |

^*-^  [Chi-square test- Evaluation of the psychiatric symptoms (depression, anxiety and stress) depending on the type of bariatric surgery performed for morbid obesity in Poland and Germany](https://www.google.com/url?sa=t&rct=j&q=&esrc=s&source=web&cd=&cad=rja&uact=8&ved=2ahUKEwjgrvPc89DxAhWvw4sKHTE7A-kQFjAAegQIAxAD&url=http%3A%2F%2Fwww.sthda.com%2Fenglish%2Fwiki%2Fkruskal-wallis-test-in-r&usg=AOvVaw1tv9mvoQIYVZWuw8Oh2xyT)

**Supplementary Table 2 Evaluation of the psychiatric symptoms (depression, anxiety and stress) depending on gender of patients undergoing bariatric surgery or conservative treatment for morbid obesity in Poland and Germany n=564.**

|  | **Baseline** | | | | **Follow-up** | | | |
| --- | --- | --- | --- | --- | --- | --- | --- | --- |
| **Level of depression:** | **POLAND n=354** | | **GERMANY n=210** | | **POLAND n=354** | | **GERMANY n=210** | |
|  | **Woman (%)** | **Men (%)** | **Woman (%)** | **Men (%)** | **Woman (%)** | **Men (%)** | **Woman (%)** | **Men (%)** |
| Normal | 1.46 | 8.75  p=0.0009^*^ | 37.34  p<0.0001^&^ | 46.15  p=0.2597^*^  p<0.0001^**^ | 22.99 | 37.50  p=0.016^*^ | 37.82  p=0.001^&^ | 46.15  p=0.022^*^  p=0.3233^**^ |
| Mild | 36.13 | 46.25  p=0.1016 | 17.09  p<0.0001 | 26.92  p=0.1209  p=0.0258 | 12.41 | 18.75  p=0.016 | 9.62  p=0.3793 | 13.46  p=0.035  p=0.4255 |
| Moderate | 23.72 | 10.00  p=0.0076 | 12.66  p=0.0054 | 5.77  p=0.1676  p=0.3902 | 28.83 | 23.75  p=0.016 | 17.31  p=0.0074 | 26.92  p=0.011  p=0.6811 |
| Severe | 28.83 | 25.00  p=0.5019 | 10.13  p<0.0001 | 13.46  p=0.5048  p=0.1083 | 11.68 | 7.50  p=0.016 | 12.82  p=0.7264 | 5.77  p=0.069  p=0.7000 |
| Extremely severe | 9.85 | 10.00  p=0.9685 | 22.78  p=0.0002 | 7.69  p=0.0162  p=0.6519 | 24.09 | 12.50  p=0.016 | 22.44  p=0.6968 | 7.69  p=0.035  p=0.3805 |
| **Level of anxiety:** | **Woman (%)** | **Men (%)** | **Woman (%)** | **Men (%)** | **Woman (%)** | **Men (%)** | **Woman (%)** | **Men (%)** |
| Normal | 7.30 | 15.00  p=0.0346 | 6.96  p=0.8951 | 1.92  p=0.1743  p=0.0137 | 33.21 | 55.00  p<0.001 | 42.31  p=0.0585 | 57.69  p=0.015  p=0.7609 |
| Mild | 9.85 | 11.25  p=0.7155 | 8.86  p=0.7352 | 7.69  p=0.7938  p=0.5024 | 9.49 | 16.25  p<0.001 | 60.00  p<0.001 | 40.00  p=0.016  p=0.0023 |
| Moderate | 26.64 | 33.75  p=0.214 | 15.82  p=0.0097 | 30.77  p=0.0183  p=0.7211 | 21.17 | 16.25  p<0.001 | 21.15  p=0.9961 | 19.23  p=0.187  p=0.6592 |
| Severe | 28.47 | 17.50  P=0.0490 | 26.58  p=0.6727 | 21.15  p=0.4343  p=0.601 | 14.60 | 2.50  p<0.001 | 9.62  p=0.1354 | 3.85  p=0.187  p=0.6585 |
| Extremely severe | 27.74 | 22.50  p=0.3505 | 41.77  p=0.0028 | 38.46  p=0.6737  p=0.0478 | 21.53 | 10.00  p<0.001 | 21.15  p=0.9261 | 7.69  p=0.015  p=0.6519 |
| **Level of stress:** | **Woman (%)** | **Men (%)** | **Woman (%)** | **Men (%)** | **Woman (%)** | **Men (%)** | **Woman (%)** | **Men (%)** |
| Normal | 17.52 | 22.50  p=0.3143 | 13.92  p=0.3281 | 13.46  p=0.9373  p=0.1952 | 44.53 | 67.50  p=0.004 | 46.79  p=0.6495 | 53.85  p=0.002  p=0.1142 |
| Mild | 35.40 | 36.25  p=0.8889 | 13.29  p<0.0001 | 17.31  p=0.4724  p=0.0189 | 13.87 | 11.25  p=0.004 | 11.54  p=0.4882 | 3.85  p=0.002  p=0.1329 |
| Moderate | 18.98 | 21.25  p=0.6521 | 32.28  p=0.0018 | 19.23  p=0.0722  p=0.7786 | 16.06 | 11.25  p=0.004 | 16.03  p=0.9935 | 17.31  p=0.147  p=0.3215 |
| Severe | 17.88 | 17.50  p=0.9377 | 27.85  p=0.0152 | 26.92  p=0.8956  p=0.1958 | 16.42 | 8.75  p=0.004 | 17.95  p=0.6833 | 15.38  p=0.253  p=0.2409 |
| Extremely severe | 10.22 | 2.50  p=0.0292 | 12.66  p=0.4370 | 23.08  p=0.0698  p=0.0002 | 9.12 | 1.25  p=0.004 | 7.69  p=0.6096 | 9.62  p=0.886  p=0.0241 |

*- Mann-Whitney U Test, A comparison of psychiatric symptoms (depression, anxiety, and stress) depending on gender in Poland and Germany;

**- Mann-Whitney U Test, A comparison of psychiatric symptoms (depression, anxiety, and stress) between men in Poland versus in Germany;

&- Mann-Whitney U Test, A comparison of psychiatric symptoms (depression, anxiety, and stress) between women in Poland versus in Germany.
